# Supplementary material for: Commensal consortia decolonize Enterobacteriaceae via ecological control
Source: Nature. 2024 Sep 18;633(8031):878–86. doi: 10.1038/s41586-024-07960-6 (PMC11424487; doi:10.1038/s41586-024-07960-6)
Supplement: Supplementary file 1 — This file contains Supplementary Discussion and Supplementary Figs. 1–5. [file 41586_2024_7960_MOESM1_ESM.pdf]

nature portfolio

https://doi.org/10.1038/s41586-024-07960-6

Supplementary information

Commensal consortia decolonize Enterobacteriaceae via ecological control

In the format provided by the authors and unedited

Commensal consortia decolonize *Enterobacteriaceae* via ecological control

Munehiro Furuichi[1,2,§], Takaaki Kawaguchi[1,2,§], Marie-Madlen Pust[3,4,§], Keiko Yasuma-Mitobe[1,§], Damian R. Plichta[3], Naomi Hasegawa[1], Takashi Ohya[1,2], Shakti Bhattarai[5], Satoshi Sasajima[1], Yoshimasa Aoto[6], Timur Tuganbaev[1,13], Mizuki Yaginuma[1], Masahiro Ueda[2,6], Nobuyuki Okahashi[2,7,11], Kimiko Amafuji[6], Yuuko Kiridooshi[6], Kayoko Sugita[1], Martin Stražar[3], Julian Avila-Pacheco[3], Kerry Pierce[3], Clary B. Clish[3], Ashwin N. Skelly[1], Masahira Hattori[2,8], Nobuhiro Nakamoto[9], Silvia Caballero[10], Jason M. Norman[10], Bernat Olle[10], Takeshi Tanoue[1,2], Wataru Suda[2,8], Makoto Arita[2,11,14], Vanni Bucci[5], Koji Atarashi[1,2,13], Ramnik J. Xavier[3,4,11*], Kenya Honda[1,2,13*]

1 Department of Microbiology and Immunology, Keio University School of Medicine, Tokyo, Japan.
2 RIKEN Center for Integrative Medical Sciences, Yokohama, Japan.
3 Infectious Disease and Microbiome Program, Broad Institute of MIT and Harvard, Cambridge, MA, USA.
4 Center for Computational and Integrative Biology, Massachusetts General Hospital and Harvard Medical School, Boston, MA, USA.
5 Department of Microbiology and Physiological Systems, Program in Microbiome Dynamics, UMass Chan Medical School, Worcester, MA, USA
6 JSR-Keio University Medical and Chemical Innovation Center, Keio University School of Medicine, Tokyo, Japan.
7 Department of Bioinformatic Engineering, Graduate School of Information Science and Technology, Osaka University, Osaka, Japan
8 Cooperative Major in Advanced Health Science, Graduate School of Advanced Science and Engineering, Waseda University, Tokyo, Japan
9 Division of Gastroenterology and Hepatology, Department of Internal Medicine, Keio University School of Medicine, Tokyo, Japan
10 Vedanta Biosciences, Cambridge, MA, USA
11 Division of Physiological Chemistry and Metabolism, Graduate School of Pharmaceutical Sciences, Keio University, Tokyo, Japan
12 Department of Molecular Biology, Massachusetts General Hospital, Boston, MA, USA.
13 Human Biology Microbiome Quantum Research Center (Bio2Q), Keio University, Tokyo, Japan

[§]These authors equally contributed to this work
*To whom correspondence should be addressed.
Kenya Honda; Tel: +81-3-5363-3768; Fax: +81-3-5361-7658; Email: kenya@keio.jp
Ramnik J. Xavier; Tel: 617-643-3331; Fax: 617-643-3328; Email: xavier@molbio.mgh.harvard.edu

SUPPLEMENTARY INFORMATION

Supplementary Discussion

1. F18-mix is a minimal effector consortium

To assess the contribution of each member of F18-mix to *Klebsiella* decolonization capacity, we created 7 derivative consortia by subtracting various combinations of bacterial species, yielding consortia that ranged in size from 12 to 17 strains. These derivative consortia exhibited varying capacities to decolonize Kp-2H7 *in vivo*, though none was as effective as the full F18-mix (**Fig. 1c**). Specifically, F18-mix was subdivided into four phylogenetic groups and derivative subsets lacking either group A (4 *Blautia* strains), B (6 *Lachnospiraceae* strains), C (5 Bacillota strains), or D (3 strains from other phyla) were tested for their ability to decolonize Kp-2H7. Compared to the full F18-mix, all derivative subsets exhibited reduced decolonization capacity (**Fig. 1c** and **Fig. 1f**). Because exclusion of the group D strains resulted in the greatest drop in efficacy, we additionally tested three different 17-strain mixtures, each excluding one of the three group D strains (f37_*Escherichia coli*, f35_*Fusobacterium ulcerans*, or f01_*Bifidobacterium longum*) from F18-mix. These three 17-mixes demonstrated a significantly impaired ability to decolonize Kp-2H7 as compared to the full F18-mix (**Fig. 1c** and **Supplementary Fig. 1a**). These results suggest that the F18 members act cooperatively and that all phylogenetic components are required to achieve maximal Kp-2H7 suppression. An alternate F18-mix (altF18-mix) generated by replacing f37_*E. coli* and f01_*B. longum* with F13-mix-derived f34_*Veillonella* and f09_*Parabacteroides* also exhibited significantly reduced activity (**Fig. 1c** and **Supplementary Fig. 1b**), indicating that the observed suppressive effect is not merely a function of strain number but rather depends on consortium composition. Additionally, to assess whether the contribution of our f37_*E. coli* strain to the therapeutic activity of F18-mix was strain-specific, we swapped it out for the *E. coli* Nissle1917 strain, which boasts a well-established safety profile[1]. There was no difference in decolonization capacity regardless of which *E. coli* strain was included (**Supplementary Fig. 1b**), suggesting that the therapeutically-active consortium is not necessarily limited to the strains isolated in this study and that safety concerns related to clinical translation can potentially be mitigated by selecting strains that exhibit similar functionalities and have established safety profiles.

2. Competition of F18-mix with commensals and pathobionts

The F18-mix preferentially uses gluconate as one of its primary carbohydrate sources, effectively competing with *Enterobacteriaceae*. This competitive dynamic likely extends to commensal bacteria as well. Indeed, bacterial species such as *Bifidobacterium*, *Ruminococcus*, and *Megasphaera*, which also possess genes for the gluconate metabolism pathway (**Extended Data Fig. 11**), were outcompeted by the F18-mix, as demonstrated in experiments involving 7 commensals, the I41-mix, and the K46-mix (**Extended Data Fig. 3e-g**). Particularly, i01_*B. adolescentis* strain, which carries gluconate kinase operon genes and preferentially utilizes gluconate (**Extended Data Fig. 11** and **Supplementary Fig. 2**), showed reduced abundance after introduction of the F18-mix (**Extended Data Fig. 3e-g**), likely due to metabolic competition. Therefore, the interaction between the F18-mix and commensal bacteria must be more extensively evaluated when applying the F18-mix in clinical settings.

In the analysis of the metagenome datasets from IBD cohorts, by focusing on MSPs that carry genes associated with gluconate metabolism pathways, we identified a significant association of

Citrobacter, *Megasphaera*, *Megamonas, E. coli*, and *Klebsiella* species with disease activity (**Fig. 4d**). *E. coli* and *Klebsiella* have been consistently identified in previous metagenome-based association studies. However, species such as Citrobacter, Megasphaera, and Megamonas have not been as clearly differentiated through simple metagenome comparisons between IBD and non-IBD cohorts. These potential pathobionts could be targets for suppression by F18-mix-mediated gluconate deprivation. Our findings provide crucial insights for future IBD research, highlighting the importance of targeted microbial analysis and the incorporation of niche competition in understanding disease mechanisms.

3. Role of GntK and GntR in gluconate and glucosamine metabolism

Kp-2H7 *gntR* transposon insertion mutants demonstrated dominance *in vivo* in mice colonized with Kp-TPs and Kp-TPs+F13-mix, rapidly outcompeting other mutant strains. In contrast, their abundance rapidly and significantly declined when F18-mix was introduced (**Fig. 3c** and **Extended Data Fig. 10c**). To unravel the molecular basis of this phenomenon, we generated Δ*gntR* and Δ*gntK* Kp-2H7 strains (**Supplementary Fig. 3**) and conducted RNA-seq analysis on wild-type (WT), Δ*gntR*, and Δ*gntK* Kp-2H7 strains cultured in minimal medium supplemented with either glucose or gluconate. Unexpectedly, the WT strain grown in gluconate-supplemented medium exhibited marked upregulation of genes predicted to be involved in glucosamine metabolism, whereas the Δ*gntR* mutant did not (**Extended Data Fig. 10d**). These findings suggest that, in addition to its role in negatively regulating gluconate operon genes, GntR is also involved in positively regulating glucosamine metabolism. This hypothesis is consistent with prior studies showing that GntR functions as a positive regulator of genes involved in citrate fermentation, glycoside hydrolase family genes, β-phosphoglucomutase, and unlinked sugar phosphotransferase in *Enterococcus* species[2,3]. Interestingly, the Δ*gntK* and Δ*gntR* strains grew poorly compared to WT in glucosamine-supplemented media (**Extended Data Fig. 10b**), indicating that both genes are involved in glucosamine utilization in addition to gluconate metabolism.

While the complete glucosamine metabolic pathway in microbes has not been fully characterized, several key steps have been inferred based on predicted gene functions within the glucosamine operon: the conversion of glucosamine to glucosaminate-6P by DgaABCD, then to 2-keto-3-deoxygluconate-6P (KDG-6P) by DgaE, and ultimately to pyruvate by DgaF (Eda) [4]. It is likely that GntR plays a key role in positively regulating this pathway genes. Notably, the enzyme responsible for the initial glucosamine phosphorylation is not found in the putative Kp-2H7 glucosamine operon, suggesting that GntK might perform this function.

The markedly different response of *gntR* transposon insertion mutants to F13-mix versus F18-mix *in vivo* might be linked to a hierarchy of *Klebsiella* carbohydrate preferences. Gluconate is a preferred carbon source for *Klebsiella*, and when it is abundant (such as in Kp-2H7 monocolonization or F13-mix co-colonization conditions) the lack of GntR-mediated repression causes gluconate operon gene upregulation, thereby enhancing gluconate utilization and conferring a competitive growth advantage over other mutants. Under such conditions, the dependency on glucosamine as an energy source becomes negligible, allowing robust growth of *gntR* mutant strains. However, in the presence of species capable of efficiently utilizing gluconate like F18-mix, upregulation of gluconate operon genes in *gntR* mutants becomes futile. As gluconate levels dwindle due to concurrent consumption by *Klebsiella* and F18-mix, *Klebsiella* may be forced to shift toward utilizing less-preferred carbon sources such as glucosamine. In these circumstances, GntR-mediated enhancement of glucosamine

utilization becomes crucial to growth and survival, and as such *gntR* mutant strains is outcompeted and suppressed.

4. Hierarchy of *Klebsiella* carbohydrate preferences and importance of gluconate metabolism

Supplementation of a gluconate-deficient AIN93G diet with glucosamine significantly increased Kp-2H7 levels *in vivo* (**Extended Data Fig. 10a**). Thus, competition for glucosamine may contribute to the effect of F18-mix. However, this may depend on the condition of gluconate depletion in the milieu, such as when F18-mix is present or the diet is deficient in gluconate. Indeed, unlike gluconate, which is both host- and diet-derived, glucosamine is almost exclusively diet-derived and is present at levels approximately 20 times lower than gluconate in the intestine (**Extended Data Fig. 9a**). Therefore, under steady-state conditions, it is more plausible that *Klebsiella* predominantly relies on gluconate as a crucial energy source for growth in the gut, and that the F18-mix competes for this resource. A similar observation was made with galactitol. Supplementation of a gluconate-deficient AIN93G diet with galactitol led to a significant increase in Kp-2H7 levels (**Extended Data Fig. 10a**). However, faecal levels of galactitol were below the detection limit (**Extended Data Fig. 9b, 10b**), and therefore, it is unlikely that competition for galactitol plays a major role in the F18-mix-mediated suppression of *Klebsiella*. Similarly, supplementation of a gluconate-deficient AIN93G diet with sorbitol led to a significant increase in Kp-2H7 levels (**Extended Data Fig. 10a**). However, despite high intestinal levels (**Fig. 3f** and **Extended Data Fig. 9b**), sorbitol could not compensate for the restricted availability of gluconate for *Klebsiella*. This is evidenced by the fact that F18-mix decolonizes Kp-2H7 despite having less-efficient sorbitol metabolism (**Extended Data Fig. 9b**). However, when gluconate was excluded from the diet and its levels further depleted by the F18-mix, supplementing the diet with sorbitol became effective in increasing Kp-2H7 levels *in vivo* (**Extended Data Fig. 10a**). Therefore, all data support the notion that, while competition for glucosamine, galactitol, and sorbitol may contribute mechanistically, it is more plausible that *Klebsiella* relies predominantly on gluconate as a crucial energy source for growth in the gut and that the F18-mix competes for this resource.

While gluconate is clearly one preferred carbon source that can affect *Klebsiella* growth and fitness, its significance may extend beyond simply serving as a nutrient. The availability and metabolism of gluconate could have cascading effects on microbial interaction patterns in the intestine. It is possible that gluconate metabolism may induce phenotypic changes in *Klebsiella* or alter the intestinal environment by promoting changes in pH levels, producing inhibitory substances, or modulating signalling pathways, for example. Such changes could potentially impact the ability of *Klebsiella* to utilize other carbohydrates, such as sorbitol and glucosamine, as effective energy sources for growth and survival. Further research is required to fully understand these complex metabolic regulatory mechanisms in the gut.

Supplementary References

1 Crook, N. *et al.* Adaptive Strategies of the Candidate Probiotic E. coli Nissle in the Mammalian Gut. *Cell Host Microbe* **25**, 499-512 e498 (2019). https://doi.org/10.1016/j.chom.2019.02.005

2 Blancato, V. S., Repizo, G. D., Suarez, C. A. & Magni, C. Transcriptional regulation of the citrate gene cluster of Enterococcus faecalis Involves the GntR family transcriptional activator CitO. *J Bacteriol* **190**, 7419-7430 (2008). https://doi.org/10.1128/JB.01704-07

3 Van Tyne, D. *et al.* Impact of antibiotic treatment and host innate immune pressure on enterococcal adaptation in the human bloodstream. *Sci Transl Med* **11** (2019). https://doi.org/10.1126/scitranslmed.aat8418

4 Miller, K. A., Phillips, R. S., Mrazek, J. & Hoover, T. R. Salmonella utilizes D-glucosaminate via a mannose family phosphotransferase system permease and associated enzymes. *J Bacteriol* **195**, 4057-4066 (2013). https://doi.org/10.1128/JB.00290-13

Supplementary Figures

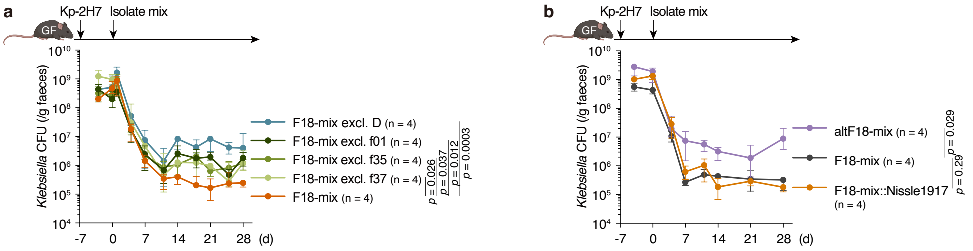

Supplementary Fig. 1. Impaired decolonization capacity of 17-mix and altF18-mix compared to full F18-mix. GF B6 mice were monocolonized with Kp-2H7 and subsequently treated with the indicated F18-mix-derived bacterial mix. In panel (**a**), three different 17-strain mixtures, each excluding one of the three group D strains (f37_*Escherichia coli*, f35_*Fusobacterium ulcerans*, or f01_*Bifidobacterium longum*) from the F18-mix, were tested. These three 17-mixes demonstrated a significantly impaired ability to decolonize Kp-2H7 as compared to the full F18-mix. In panel (**b**), an alternate F18-mix (altF18-mix) created by replacing f37_*E. coli* and f01_*B. longum* with strains f34_*Veillonella* and f09_*Parabacteroides* from F13-mix was tested. The altF18-mix also exhibited significantly reduced activity. F18-mix::Nissle1917 indicates F18-mix with the f37_*E. coli* strain swapped out for *E. coli* Nissle1917. Data are expressed as median ± IQR, representative of two independent experiments. Statistical analysis was performed using the Kruskal-Wallis test with the Benjamini-Hochberg correction for multiple comparisons at day 28 compared to F18-mix.

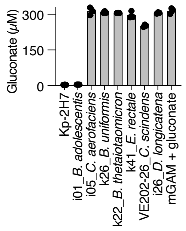

Supplementary Fig. 2. Gluconate consumption by Bifidobacterium. Gluconate consumption was evaluated by incubating each of the seven indicated commensal strains and Kp-2H7 individually in mGAM broth containing 300 μM gluconate for 48 hours (n = 3 biological replicates). The resulting gluconate concentration in the culture supernatant was measured by LC-MS/MS.

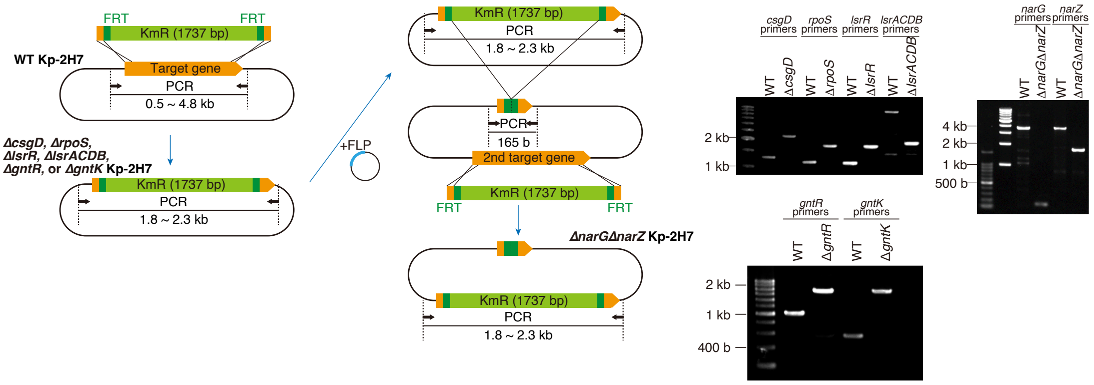

Supplementary Fig. 3. Generation of gene deletion mutants of Kp-2H7.
The schematic illustrates the generation of Kp-2H7 deletion mutants using the pRED/ET system. Kp-2H7 cells were transformed with the linear DNA fragment (the FRT-flanked kanamycin-resistance cassette) to generate Δ*csgD*, Δ*rpoS*, Δ*lsrR*, Δ*lsrACDB*, Δ*narG*, Δ*gntK*, and Δ*gntR* Kp-2H7 strains.
To generate the Δ*narG*Δ*narZ* double knockout strain, the KmR cassette was removed from Δ*narG* Kp2H7 using the FLP-recombinase expression plasmid. Subsequently, the FRT-flanked kanamycin-resistance cassette was transformed again into the *narZ* gene locus.
PCR validation for the indicated mutants was conducted. Representative images from two independent experiments with similar results are shown. Primers used for the PCR validation are listed in Table S9.
KmR, kanamycin resistance; FRT, flippase recognition target; FLP, flippase.

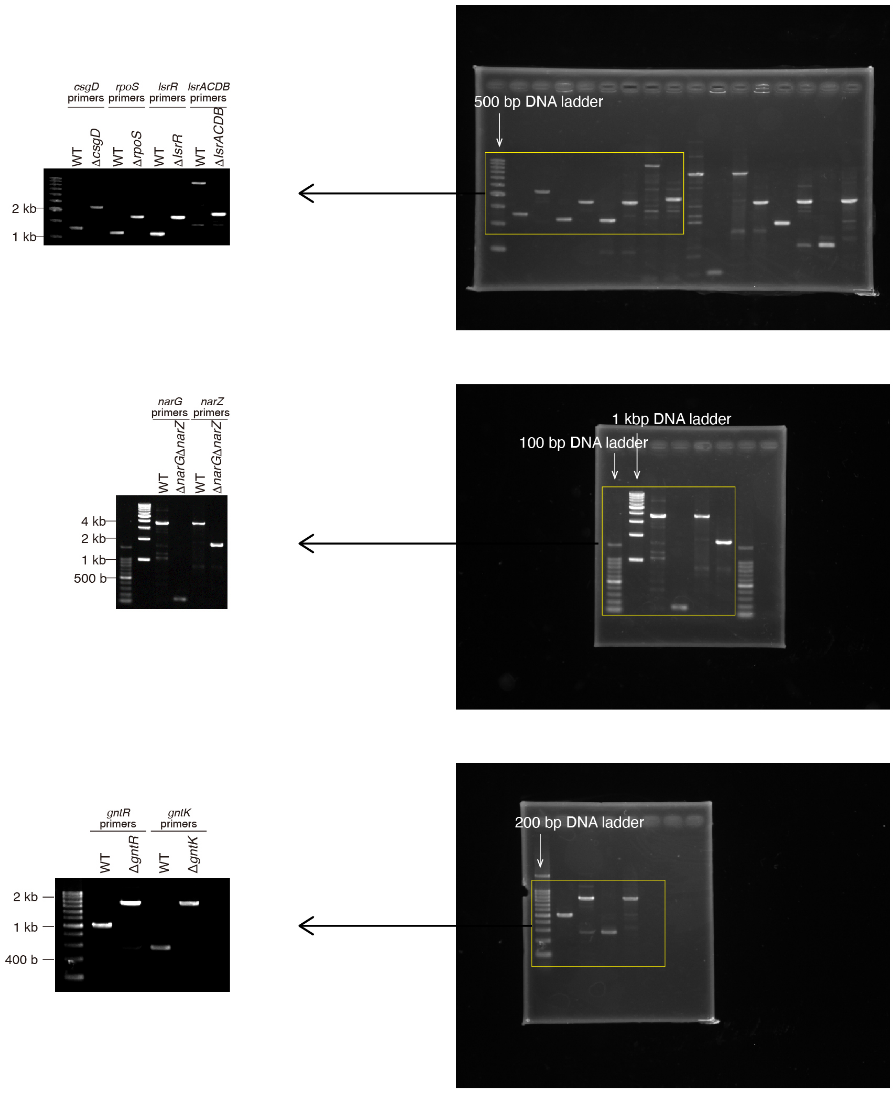

Supplementary Fig. 4. Uncropped agarose gel images from Supplementary Fig. 3.
Boxes with yellow lines represent the cropped regions used in the figures.

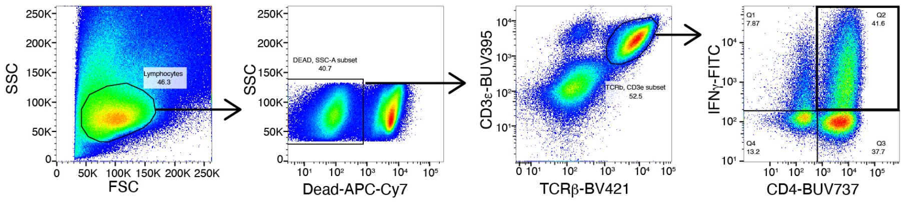

Supplementary Fig. 5.
Gating strategy to determine the frequency of IFNγ+ cells among colonic lamina prpopria in CD4+TCRβ+ T cells. (Extended Data Fig. 4g)
