# Supplementary material for: Commensal consortia decolonize Enterobacteriaceae via ecological control
Source: Nature. 2024 Sep 18;633(8031):878–86. doi: 10.1038/s41586-024-07960-6 (PMC11424487; doi:10.1038/s41586-024-07960-6)
Supplement: Supplementary file 2 — Reporting Summary [file 41586_2024_7960_MOESM2_ESM.pdf]

nature portfolio

Corresponding author(s): Kenya Honda

Last updated by author(s): Jul 29, 2024

Reporting Summary

Nature Portfolio wishes to improve the reproducibility of the work that we publish. This form provides structure for consistency and transparency in reporting. For further information on Nature Portfolio policies, see our Editorial Policies and the Editorial Policy Checklist.

Statistics

For all statistical analyses, confirm that the following items are present in the figure legend, table legend, main text, or Methods section.

n/a	Confirmed	
☐	☒	The exact sample size (*n*) for each experimental group/condition, given as a discrete number and unit of measurement
☐	☒	A statement on whether measurements were taken from distinct samples or whether the same sample was measured repeatedly
☐	☒	The statistical test(s) used AND whether they are one- or two-sided<br>*Only common tests should be described solely by name; describe more complex techniques in the Methods section.*
☐	☒	A description of all covariates tested
☐	☒	A description of any assumptions or corrections, such as tests of normality and adjustment for multiple comparisons
☐	☒	A full description of the statistical parameters including central tendency (e.g. means) or other basic estimates (e.g. regression coefficient) AND variation (e.g. standard deviation) or associated estimates of uncertainty (e.g. confidence intervals)
☐	☒	For null hypothesis testing, the test statistic (e.g. *F*, *t*, *r*) with confidence intervals, effect sizes, degrees of freedom and *P* value noted<br>*Give P values as exact values whenever suitable.*
☒	☐	For Bayesian analysis, information on the choice of priors and Markov chain Monte Carlo settings
☒	☐	For hierarchical and complex designs, identification of the appropriate level for tests and full reporting of outcomes
☐	☒	Estimates of effect sizes (e.g. Cohen's *d*, Pearson's *r*), indicating how they were calculated

Our web collection on statistics for biologists contains articles on many of the points above.

Software and code

Policy information about availability of computer code

Data collection	LC-MS/MS data were obtained using Analyst software version 1.7.1.<br>Light Cycler 480 1.5.1 software was used for collecting qPCR data.<br>16S rRNA gene sequencing was sequenced using PacBio Revio system. Bacterial whole-genome was sequenced using the Illumina MiSeq and PacBio Sequel.<br>RNA sequence was performed using NovaSeq 6000 (Illumina Inc.) HiSeq X (Illumina Inc.).<br>Tn-seq was performed using HiSeq 2500 (Illumina Inc.).
Data analysis	For 16S rRNA gene sequencing, the sequenced reads were uploaded to the DADA2 (version 1.30.0) in R (version 4.3.3) to construct ASVs using the filterAndTrim function. FL16s-ASVs were then subjected to a homology search against 16S rRNA gene sequences extracted from publicly available genomes (downloaded from GenBank on September 12, 2023) using BLASTN with a maximum e-value cut-off of 1e-10. Top hits were determined by the highest bitscore.<br>Bacterial whole-genome sequencing were assembled using Unicycler. Taxonomic assignment of the genomes was determined by classify_wf of GTDB-tk version 2.3.0 with GTDB database R214. NCBI taxonomy of fastANI reference genome related to the genome of each strain was retrieved using NCBI-genome-download version 0.3.3 (ncbi-genome-download; DOI: 10.5281/zenodo.8192432) and rankedlineage.dmp from NCBI taxonomy database (downloaded on 14/09/2023).<br>Transcriptome analysis was performed using Trimmomatic version 0.39, FASTX-Toolkit version 0.0.13, bowtie2 version 2.3.4.1. (in vivo) or 2.4.4 (in vitro), featureCounts3 version 2.0.1., STAR version 2.7.2b, and DESeq274 version 1.28.1 (in vivo) or 1.30.1 (in vitro).<br>Tn-seq was performed using Trimmomatic version 0.39, FASTX-Toolkit version 0.0.13, minimap2 version 2.17-r941, bowtie2 version 2.4.2, and featureCounts3 version 1.5.2.

For manuscripts utilizing custom algorithms or software that are central to the research but not yet described in published literature, software must be made available to editors and reviewers. We strongly encourage code deposition in a community repository (e.g. GitHub). See the Nature Portfolio guidelines for submitting code & software for further information.

Data

Policy information about availability of data

All manuscripts must include a data availability statement. This statement should provide the following information, where applicable:

- Accession codes, unique identifiers, or web links for publicly available datasets
- A description of any restrictions on data availability
- For clinical datasets or third party data, please ensure that the statement adheres to our policy

The data of Tn-Seq data and RNA sequence data of host, Kp in vivo, and Kp in vitro are deposited in the DNA Data Bank of Japan under BioProject PRJDB17114. Genome sequences of the 31, 41, and 46 strains isolated from donor F, I and K, respectively are deposited in the DNA Data Bank of Japan under BioProject PRJDB17661.

Research involving human participants, their data, or biological material

Policy information about studies with human participants or human data. See also policy information about sex, gender (identity/presentation), and sexual orientation and race, ethnicity and racism.

Reporting on sex and gender	The outcomes were consistent across sexes. Therefore, we have chosen not to delineate the results by these factors to avoid undue emphasis on distinctions that were not meaningful in the context of our study.
Reporting on race, ethnicity, or other socially relevant groupings	In this study, no distinction of race or ethnicity was made at any point in the collection of human faecal samples or any other analysis.
Population characteristics	Human faecal samples were obtained from healthy human donors (n=5). The median age of healthy human was 33 years (range: 30-41), and all lf them were Japanese.
Recruitment	Human faecal samples were collected at Keio University and Keio University Hospital. Informed consent was obtained from each subject.
Ethics oversight	Human faecal samples were obtained from healthy human donors following the protocol approved by the Institutional Review Board of Keio University School of Medicine (approval numbers #20150075, #20140211, and #20150075).

Note that full information on the approval of the study protocol must also be provided in the manuscript.

Field-specific reporting

Please select the one below that is the best fit for your research. If you are not sure, read the appropriate sections before making your selection.

☒ Life sciences ☐ Behavioural & social sciences ☐ Ecological, evolutionary & environmental sciences

For a reference copy of the document with all sections, see nature.com/documents/nr-reporting-summary-flat.pdf

Life sciences study design

All studies must disclose on these points even when the disclosure is negative.

Sample size	No statistical methods were used to predetermine sample size. Sample size for animal experiments were chosen according to preliminary pilot studies, balancing statistical robustness and animal welfare.
Data exclusions	No data was excluded
Replication	For all the experiments, reproducibility was verified by conducting the experiment at least twice, which yielded comparable results.
Randomization	In animal experiments, samples were randomly allocated into experimental groups. Sex-matched littermates were used and the experiments were designed to test a single variable.
Blinding	The CFU counting and LC-MS/MS analysis were performed by technicians who were blinded to expected outcomes. The histological analysis were evaluated blind by two investigators. All other studies were not strictly blinded because the measurements were based on objective analysis methods.

Reporting for specific materials, systems and methods

We require information from authors about some types of materials, experimental systems and methods used in many studies. Here, indicate whether each material, system or method listed is relevant to your study. If you are not sure if a list item applies to your research, read the appropriate section before selecting a response.

Materials & experimental systems

n/a	Involved in the study
☐	☒ Antibodies
☒	☐ Eukaryotic cell lines
☒	☐ Palaeontology and archaeology
☐	☒ Animals and other organisms
☒	☐ Clinical data
☒	☐ Dual use research of concern
☒	☐ Plants

Methods

n/a	Involved in the study
☒	☐ ChIP-seq
☐	☒ Flow cytometry
☒	☐ MRI-based neuroimaging

Antibodies

Antibodies used

anti-CD3e (BUV395; BD Biosciences, 1:1000), CD4 (BUV737; BD Biosciences, 1:1000), TCRβ (BV421; Biolegend, 1:1000), and IFN-γ (FITC; Biolegend, 1:1000)

Validation

All the antibodies are commercially available. Quality validations were performed by each manufacturer. Validation statements are provided on the manufacturer's website.
anti-CD3e (BUV395; BD Biosciences) (https://www.bdbiosciences.com/en-us/products/reagents/flow-cytometry-reagents/research-reagents/single-color-antibodies-ruo/buv395-hamster-anti-mouse-cd3e.563565)
CD4 (BUV737; BD Biosciences) (https://www.bdbiosciences.com/en-us/products/reagents/flow-cytometry-reagents/research-reagents/single-color-antibodies-ruo/buv737-rat-anti-mouse-cd4.612843)
TCRβ (BV421; Biolegend) (https://www.biolegend.com/ja-jp/products/brilliant-violet-421-anti-mouse-tcr-beta-chain-antibody-7251)
IFN-γ (FITC; Biolegend) (https://www.biolegend.com/ja-jp/products/fitc-anti-mouse-ifn-gamma-antibody-995)

Animals and other research organisms

Policy information about studies involving animals; ARRIVE guidelines recommended for reporting animal research, and Sex and Gender in Research

Laboratory animals

C57BL/6 mice, maintained under specific-pathogen-free or germ-free (GF) conditions, were purchased from Sankyo Laboratories Japan, SLC Japan, or CLEA Japan. GF and gnotobiotic mice were bred and maintained within the gnotobiotic facility of Keio University School of Medicine or the JSR-Keio University Medical and Chemical Innovation Center. Il10-/- and Ifngr1-/- mice were purchased from Jackson Laboratories. Myd88-/-Trif-/- and Rag2-/-γc-/- mice were purchased from Oriental Bio Service, Japan. All animals were maintained under a 12-h light–dark cycle. A temperature of 20–24 °C and a humidity of 40–60% were used for the housing conditions.

Wild animals

The study did not involve wild animals.

Reporting on sex

We did not categorize the sexes because the differences between the sexes did not affect the results of the experiment.

Field-collected samples

The study did not involve samples collected from the field.

Ethics oversight

All animal experiments were approved by the Keio University Institutional Animal Care and Use Committee.

Note that full information on the approval of the study protocol must also be provided in the manuscript.

Plants

Seed stocks

Report on the source of all seed stocks or other plant material used. If applicable, state the seed stock centre and catalogue number. If plant specimens were collected from the field, describe the collection location, date and sampling procedures.

Novel plant genotypes

Describe the methods by which all novel plant genotypes were produced. This includes those generated by transgenic approaches, gene editing, chemical/radiation-based mutagenesis and hybridization. For transgenic lines, describe the transformation method, the number of independent lines analyzed and the generation upon which experiments were performed. For gene-edited lines, describe the editor used, the endogenous sequence targeted for editing, the targeting guide RNA sequence (if applicable) and how the editor was applied.

Authentication

Describe any authentication procedures for each seed stock used or novel genotype generated. Describe any experiments used to assess the effect of a mutation and, where applicable, how potential secondary effects (e.g. second site T-DNA insertions, mosiacism, off-target gene editing) were examined.

Flow Cytometry

Plots

Confirm that:

☒ The axis labels state the marker and fluorochrome used (e.g. CD4-FITC).

☒ The axis scales are clearly visible. Include numbers along axes only for bottom left plot of group (a 'group' is an analysis of identical markers).

☒ All plots are contour plots with outliers or pseudocolor plots.

☒ A numerical value for number of cells or percentage (with statistics) is provided.

Methodology

Sample preparation	Lymphocytes were collected from the large intestines using the method described below. The intestines were dissected longitudinally and washed with PBS to remove all luminal contents. All samples were incubated in 15 mL of Hanks' balanced salt solution (HBSS) containing 5 mM EDTA for 20 min at 37°C in a shaking water bath to remove epithelial cells. Thereafter, after removal of any remaining epithelial cells, muscular layers and fat tissues using forceps, the samples were cut into small pieces and incubated in 10 mL of RPMI1640 containing 4% foetal bovine serum (FBS), 0.5 mg/mL collagenase D (Roche Diagnostics), 0.5 mg/mL dispase II (Roche Diagnostics), and 40 µg/mL DNase I (Roche Diagnostics) for 50 min at 37°C in a shaking water bath. Thereafter, the resultant digested tissues were washed with 10 mL of HBSS containing 5 mM EDTA, resuspended in 5 mL of 40% Percoll (GE Healthcare), and underlaid with 2.5 mL of 80% Percoll in a 15-mL Falcon tube. Percoll gradient separation was performed by centrifugation at 850 × g for 25 min at 25°C. Lymphocytes were collected from the interface of the Percoll gradient and washed with RPMI1640 containing 10% FBS, and then stimulated with 50 ng/mL PMA and 750 ng/mL ionomycin (both from Sigma) in the presence of Golgistop (BD Biosciences) at 37°C for four hours.
Instrument	BD LSRFortessa (BD Biosciences)
Software	Flowjo software (TreeStar)
Cell population abundance	The Purity was determined by running a purity check of the sorted populations after the sort was completed.
Gating strategy	All samples were initially gated using forward scatter and side scatter to identify events corresponding to cells, and then using forward scatter height vs. area to enrich for single cells, next alive cells were selected by negativity for viability dye. TCD4+ T cells were defined as a CD4+ TCRβ+ CD3e+ subset within the live lymphocyte gate.

☒ Tick this box to confirm that a figure exemplifying the gating strategy is provided in the Supplementary Information.
